# Case series of Creutzfeldt-Jakob disease in a third-level hospital in Quito

**DOI:** 10.1186/s12883-018-1061-0

**Published:** 2018-04-27

**Authors:** Germaine Eleanor Torres Herrán, Andrés Damián Ortega Herrera, Braulio Martinez Burbano, Marcos Serrano-Dueñas, María Angélica Ortiz Yepez, Raúl Alberto Barrera Madera, Luis Alfredo Masabanda Campaña, Guillermo David Baño Jiménez, Denny Maritza Santos Saltos, Edgar Patricio Correa Díaz

**Affiliations:** 1 Hospital Carlos Andrade Marín, Av. 18 de Septiembre y Ayacucho, Quito, Ecuador; 2 Universidad Central del Ecuador, Calle Iquique y Sodiro, Quito, Ecuador; 3 Facultad de Medicina de la Pontifica Universidad Católica del Ecuador, Avenida 12 de Octubre y Vicente Ramón Roca, Quito, Ecuador

**Keywords:** Creutzfeldt-Jakob disease, Prion protein, Rapidly progressive dementia, 14–3-3 protein, Tau protein, RT-QuIC

## Abstract

**Background:**

Creutzfeldt-Jakob disease is a rare and fatal neurodegenerative disorder that affects mammals and humans. The prevalence of this disease in the United States is 0.5 to 1 per million inhabitants. So far in Ecuador, we do not know what the prevalence or incidence is, and only one case report has been written.

**Case presentation:**

We present a case series of Creutzfeldt-Jakob disease in a third-level hospital in Quito. The average age of symptom onset in our patients was 58.8 years. The male to female ratio was 1:1. Two patients began with cognitive/behavioral symptoms, while 4 patients began with focal neurological signs; 1 case with ataxia, 2 with gait disorders and 1 with vertigo and headache. All of the patients had the clinical features established by the World Health Organization. In addition, the entire cohort was positive for the 14–3-3 protein in cerebrospinal fluid, and had high signal abnormalities in caudate and putamen nucleus in DWI and FLAIR IRM. Only in one case, did we reach a definitive diagnosis through a pathological study. All other cases had a probable diagnosis. In this series of cases, 6 out of 6 patients died. The average time from the onset of the symptoms to death in this cohort was 13 months.

**Conclusion:**

This is the first report of a series of cases of Creutzfeldt-Jakob disease in Quito. Although definitive diagnosis must be histopathological, there are ancillary tests currently available that have allowed us to obtain a diagnosis of the disease.

## Background

Creutzfeldt-Jakob disease (CJD) is a fatal neurodegenerative disorder that affects humans. The pathophysiological mechanism of the disease consists of the formation of an abnormal isoform of the prion protein (PrP) called scrapie (PrPSc) which accumulates in the gray matter of the brain and is partially resistant to the action of proteases [1–7].

The estimated incidence of the disease in the United States is 0.5 to 1 per million inhabitants per year. The age of onset is between 55 and 75 years, both sexes are equally affected [3, 4, 6, 8, 9]. There are 4 subtypes of CJD: sporadic, genetic, iatrogenic and variant. Sporadic CJD (sCJD) is the most common form of the disease, accounting for 85–90% of all CJD cases, followed by genetic subtypes such as Gertsmann-Sträussler-Scheinker, fatal familial insomnia and familial CJD (fCJD) which are present in 10–15% of cases. Variant and iatrogenic CJD subtypes are the least frequent, representing 5% of cases [2, 7, 9]. The prognosis of the disease is fatal because 90% of patients die within the first year of symptom onset [9].

Clinical presentation of CJD is highly variable; most cases have a subacute course. Frequent manifestations include: rapidly progressive dementia associated with neuropsychiatric manifestations, cerebellar ataxia, visual symptoms, myoclonus, akinetic mutism, and pyramidal and/or extrapyramidal signs [8–10]. Atypical manifestations have also been described, which at their onset, resemble cerebrovascular disease, depression or supranuclear palsy [10]. Pre-mortem diagnosis is based on 5 types of paraclinical tests: electroencephalogram (EEG), cerebrospinal fluid (CSF) biomarkers, brain magnetic resonance imaging (MRI), positive real-time quaking-induced conversion (RT-QuIC) in CSF or other tissues and brain biopsy [11–14]. The gold standard for definitive diagnosis Of CJD is histopathological confirmation through a brain biopsy or autopsy [12–16].

## Case presentation

Ecuador has an approximate population of 16 million inhabitants of which 9,271,362 are affiliated with social security [17, 18]. The Carlos Andrade Marín Hospital (CAMH) in the city of Quito is a third-level hospital and a reference center in the country that attends nearly 600,000 patients each year [18]. One thousand two hundred patients with dementias were treated between 2012 and 2016, 60% (720 patients) were for Alzheimer’s disease and 8.58% (103 patients) for rapidly progressive dementias (DRP). Of the DRP 1.9% (2/103) were autoimmune encephalopathies, 12.6% (13/103) infectious, 48.53% (50/103) metabolic, 2.9% toxic (3/103), 8.7% vascular (9/103), 6.8% neoplastic (7/103), 4.85% primary dementias with atypical onset and 5.85% (6/103) with CJD.

In Ecuador, there are no studies of the prevalence or incidence of CJD so far, and one clinical case-report of CJD has been described [19]. We present 6 case reports of CJD diagnosed between January 2012 and June 2016 at CAMH.

### Case 1

Male patient, 48 years old, mestizo. His past medical history was non-contributory. The symptoms began in February 2012. Initial symptoms were vertigo, ataxia and gait disorder. One month later, he presented dysarthria, postural tremor of the upper limbs, headache, fluctuating episodes of disorientation and nystagmus. At 2 months, he presented movement disorders characterized by generalized chorea, myoclonus and cervical dystonia. This was compounded by changes in behavior with episodes of irritability and psychomotor agitation.

**Fig. 1:**
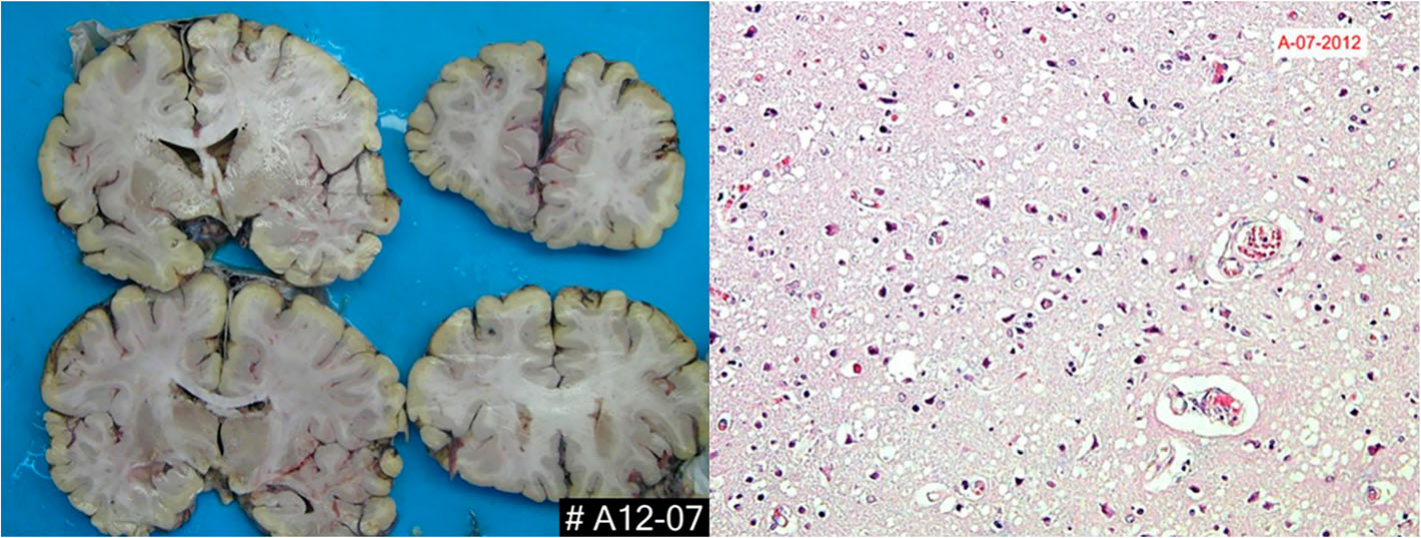
Pathologic features of prion disease in case 1. Hematoxylin and eosin (H&E) staining demonstrates typical spongiform degeneration (vacuolation) of the gray matter neuropil characteristic of Jakob-Creutzfeldt disease

Laboratory study results included normal CSF (glucose, proteins and cellularity). Tumor markers and antibodies (ANA, anti-DNA, anti-Ro, anti-LA, ANCA and anti-TPO) were negative. Body scan (CT) was negative for malignancy. The EEG demonstrated the presence of periodic sharp wave complexes, and diffusion-weighted imaging (DWI) and fluid attenuated inversion recovery (FLAIR) MRI showed high signal abnormalities in caudate and putamen nucleus. Spectroscopy MRI showed a decrease in caudate nucleus volume, as well as an increase in creatine relative to choline and a slight decrease in N-acetyl aspartate (NAA), which was concordant with neuronal loss. Under suspicion of CJD, testing of 14–3-3 protein was requested in CSF, which was positive and tau protein levels were 13.135 pg/ml. The patient died of nosocomial pneumonia. An autopsy was carried out. Macroscopic brain studies at brain autopsy revealed cerebral and cerebellar atrophy. The histopathological study showed marked neuronal loss, areas of gliosis and intracytoplasmic vacuolization of the cerebral parenchyma. PrP immunostaining was not done (Fig. 1).

### Case 2

The patient was a 74-year-old woman, mestizo, with a history of hypertension. Symptoms began in December 2013 with rapidly progressive cognitive impairment and gait disorder. A postural action tremor was also observed. At 15 days, the patient stopped recognizing her children. These symptoms were accompanied by truncal ataxia and urinary retention, followed by fluctuating periods of psychomotor agitation.

**Fig. 2:**
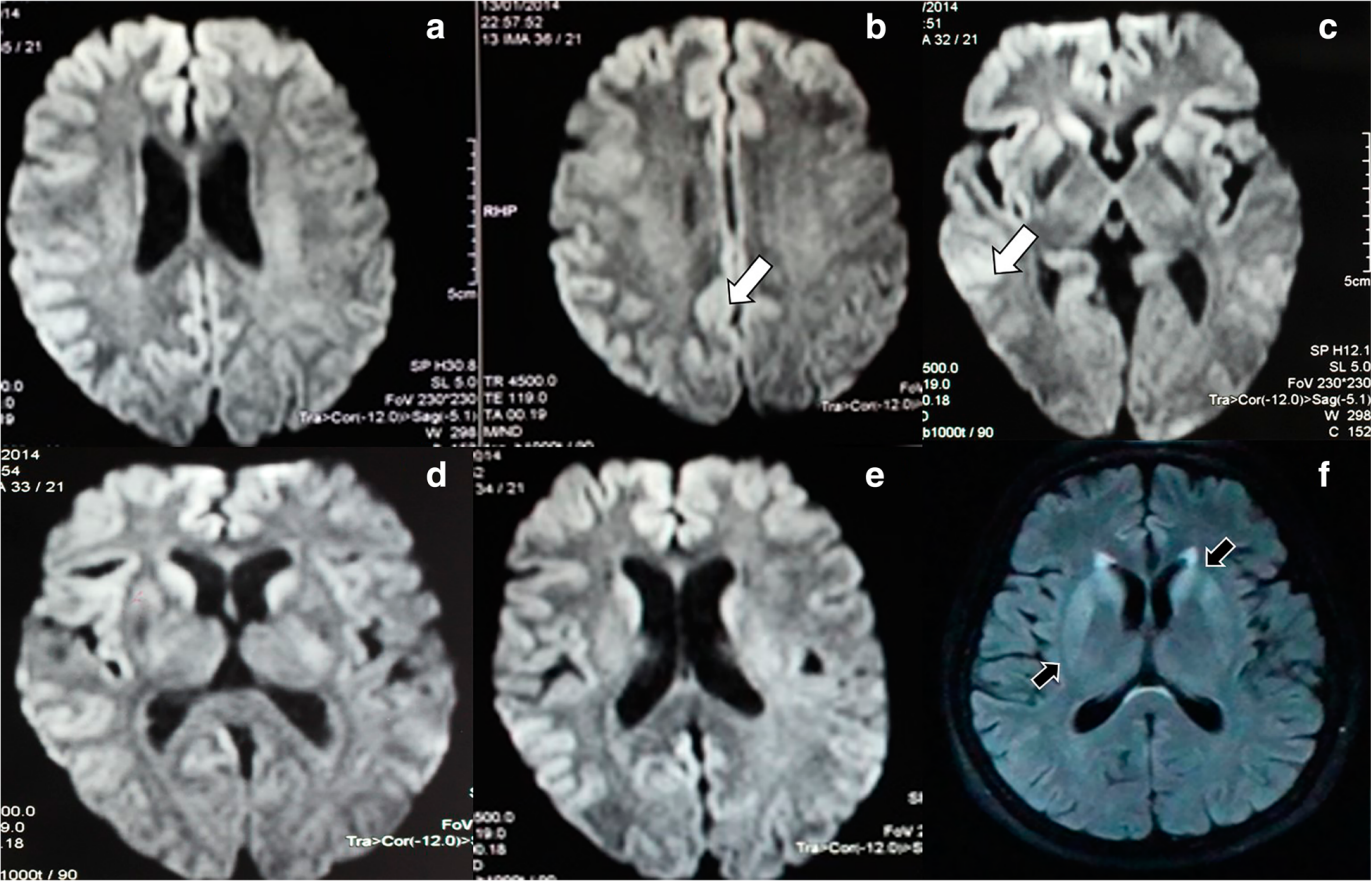
Imaging of the patient in case 2 3 months after onset of sporadic Jakob-Creutzfeldt disease. **a**-**e**, Axial diffusion-weighted imaging (DWI). Bilateral restricted diffusion cortical ribboning is shown in the bilateral temporal and parietal cortices (white arrows). **f**, fluid attenuated inversion recovery (FLAIR) shows high signal abnormalities in caudate and putamen nucleus (black arrows)

**Fig. 3:**
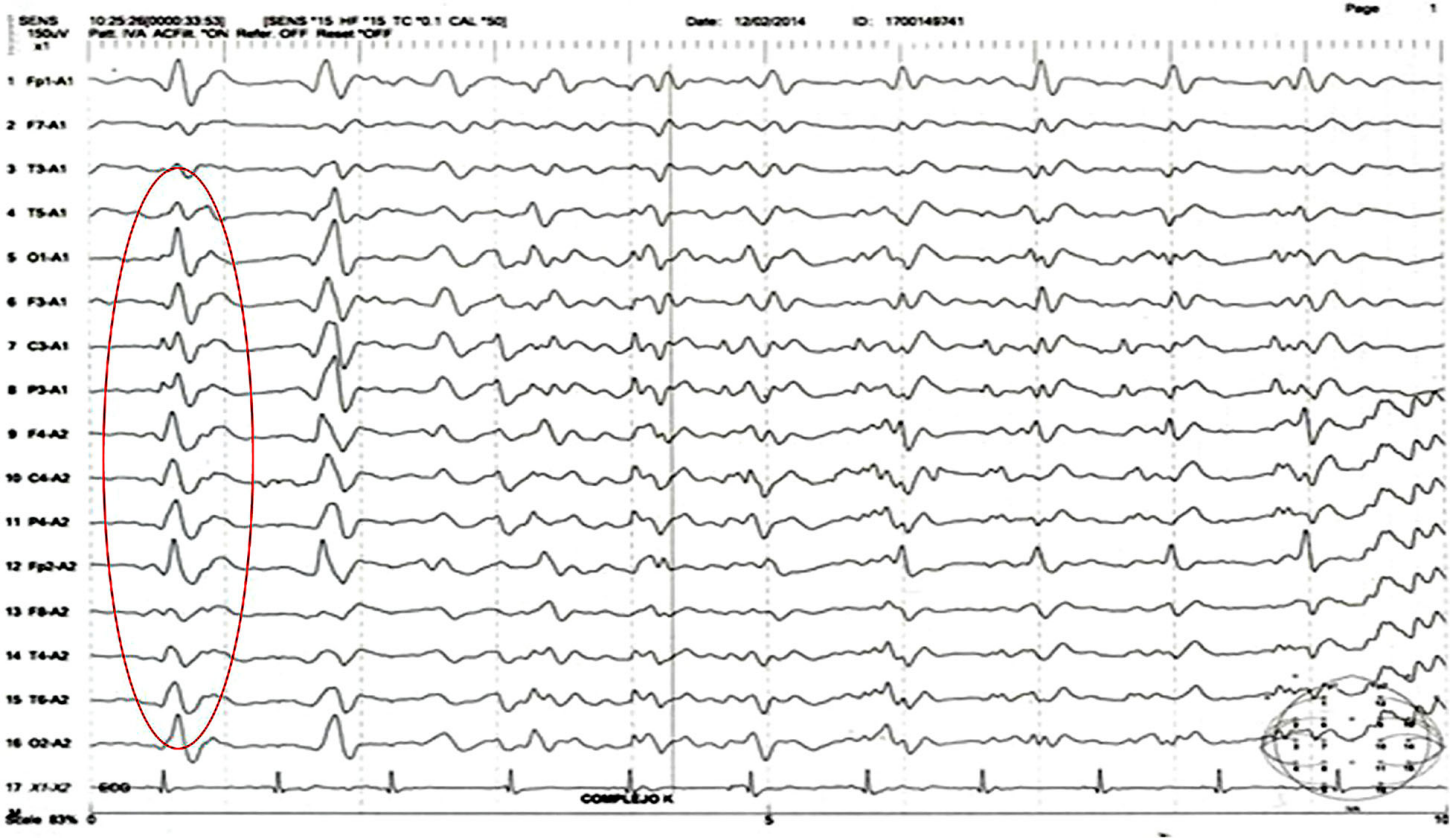
Electroencephalography of the patient in case 2 at months 3. Legend: periodic sharp wave complexes at intervals of 1 to 2 s

Laboratory studies of liver and kidney were normal, and antibody and tumor malignancy markers were negative. The study of CSF (glucose, proteins and cellularity) was normal. DWI and FLAIR MRI of the brain showed the presence of bilateral frontal and temporal cortical ribboning; high signal abnormalities in caudate and putamen nucleus was also observed (Fig. 2). At 2 months, the patient’s level of consciousness deteriorated, reaching a state of stupor. Finally, myoclonus to tactile stimuli and right hemichorea was also present. The EEG demonstrated the presence of periodic sharp wave complexes at intervals of 1 to 2 s (Fig. 3). Under suspicion of CJD, 14–3-3 protein testing in CSF was requested, and the test result was positive. The levels of tau protein in CSF were 3967 pg/ml. A diagnosis of probable sCJD was given. Family members did not authorize brain biopsy. The patient died 6 months after the onset of symptoms by nosocomial pneumonia. Autopsy was not authorized.

### Case 3

A 54-year-old male patient, mestizo, was assessed in October 2014 over the course of 4 months for sensory symptoms with paresthesia in the lower limbs and difficulty walking. At 5 months, the gait became ataxic and was accompanied by a lateropulsion on the left side. Subsequently, cognitive deterioration appeared. Problems were mainly with immediate memory and irritability. The patient also experienced visual and auditory hallucinations. A symmetrical postural tremor also appeared in the upper limbs. Initial neurological examination showed amnesic cognitive impairment, postural tremor, ataxic gait and myoclonus. His past medical history was non-contributory.

Laboratory studies were normal. Under suspicion of a paraneoplastic disease, tumor markers were tested, but the results were negative. Body CT was negative for malignancy. The study of CSF (glucose, proteins and cellularity) was normal. DWI and FLAIR MRI of the brain showed high signal abnormalities in caudate and putamen nucleus. EEG demonstrated the presence of bilateral frontotemporal paroxysmal theta activity with right-side predominance, and the neuropsychological evaluation reported a dysexecutive syndrome with severe cognitive deterioration. Under suspicion of prion disease, tau protein levels were requested, which were 3.000 pg/ml and the testing of 14–3-3 protein in CSF was positive. A diagnosis of probable sCJD was given. The patient died 5 months after the onset of symptoms by nosocomial pneumonia. Family members did not authorize an autopsy.

### Case 4

A 57-year-old male patient, mestizo. His past medical history was non-contributory. His clinical condition began in May 2014 with diplopia, vertigo and gait disorder. One month later, a rotating nystagmus appeared, in addition to ataxia and spasticity in all four limbs. An ataxic gait, dysarthria and sporadic myoclonus were also evident. For this reason, a DWI and FLAIR MRI of the brain was requested which showed a slight generalized cortical atrophy. Two months after the onset of symptoms, the patient presented non-fluent aphasia, and myoclonus became more frequent. The study of CSF (glucose, proteins and cellularity) was normal. Under suspicion of a paraneoplastic syndrome, tumor markers were tested, but the results were negative. EEG demonstrated the presence of periodic sharp wave complexes.

The second DWI and FLAIR MRI of the brain showed high signal abnormalities in caudate and putamen nucleus, in addition to a bilateral frontal cortical ribboning. The 14–3-3 protein in CSF was present and the levels of tau protein in CSF were 2976 pg/ml. A diagnosis of probable CJD was established. The patient died 14 months after the onset symptoms due to a series of nosocomial respiratory and urinary infections. The relatives did not authorize an autopsy.

### Case 5

A 64-year-old female patient, mestizo. His past medical history was non-contributory. The symptoms began in March 2014 with moderate intensity headaches, which did not subside with the use of analgesics. The headaches were accompanied by vertigo. At 4 weeks, the patient had symptoms of depression. At 4 months, she presented global cognitive impairment. At 6 months, a symmetrical postural tremor appeared in the hands. At 7 months, there was a gait disorder with ataxia and left-side lateropulsion. At 8 months, cognitive deterioration worsened. Neuropsychological assessment showed disturbances in executive functions and declarative, working and episodic memory. This condition was compatible with a moderate dementia of predominance in the frontotemporal region.

At 9 months, there was a total loss of episodic memory. The neurological evaluation showed dysarthria, paralysis of vertical gaze and myoclonus. EEG demonstrated generalized theta activity of 5 to 6 Hz. The study of CSF (glucose, proteins and cellularity) was normal. DWI and FLAIR MRI of the brain demonstrated the presence of frontotemporal cortical hyperintensity (cortical ribboning) and high signal abnormalities in the caudate and putamen nuclei. Tests for tumor markers and antibodies were negative. Body CT was normal. With the possibility of prion disease, the presence of 14–3-3 protein in CSF was tested, which was positive. Tau protein levels were 13,000 pg/ml. A diagnosis of probable CJD was established. The patient was transferred to a clinic for patients with chronic conditions and later died due to pneumonia 14 months after the initial symptoms. The family refused performance of a brain biopsy.

### Case 6

A 56-year-old female patient, mestizo, with a history of untreated type-2 diabetes mellitus, whose symptoms began in January of 2015 with dizziness and postural vertigo with a duration of only a few seconds. However, a month later these symptoms became more frequent with increased intensity. In addition, gait ataxia appeared. At 2 months, the patient began to experience insomnia. At 3 months, there were amnesic symptoms with loss of episodic memory. There were also behavioral disorders with the patient exhibiting child-like behaviors. The patient’s language became slow. The patient also presented with myoclonus and experienced visual hallucinations. The initial MRI brain study was normal.

At 5 months, the patient’s gait ataxia worsened and hyperphagia appeared. At 6 months, there was a deterioration of the patient’s level of alertness, and the patient became drowsy. Neurological evaluation demonstrated the presence of bidirectional horizontal nystagmus and vertical gaze paralysis. Symptoms such as dysarthria and ataxia of all four extremities appeared. At 7 months, the patient presented movement disorders, including right hemichorea. Among the laboratory studies requested, CSF (glucose, proteins and cellularity) was normal. With the possibility of a paraneoplastic syndrome, a CT body scan was requested, which was normal, and tumor markers were tested, which were negative. EEG showed periodic sharp wave complexes that repeated every 1 to 2 s. Under suspicion of CJD, CSF tests were performed. The 14–3-3 protein was positive and tau protein levels were 11,770 pg/ml. DWI and FLAIR MRI of the brain demonstrated the presence of high signal abnormalities in caudate and putamen nucleus and a medial frontal and parietal cortical ribboning. A diagnosis of probable sCJD was established. The patient died in a clinic for chronic conditions due pneumonia 12 months after the initial symptoms. Family members did not authorize a brain biopsy.

All patients received symptomatic treatment which included the empirical use of selective serotonin reuptake inhibitors to treatment of depression, atypical antipsychotics to treat agitation and psychosis and clonazepam to treat severe myoclonus. None of patients had a positive family history of prion disease or RPD. Genetic analysis was not done in our patients because, molecular genetic tests are not available in Ecuador (Table 1).

**Table 1:** Patient characteristics

Age/Sex	Clinical presentation	Duration of the disease (months)	Triphasic waves in EEG	MRI findings	CSF 14–3-3	Tau protein	Autopsy	Age of death (years)
48/M	Confusion, ataxia, generalized chorea, myoclonus, blurred vision	16	Present	Hyperintensity in basal ganglia	Positive	2130 pg/ml	Positive	49
74/F	Confusion, ataxia, myoclonus, urinary incontinence and psychomotor agitation	10	Present	Hyperintensity in basal ganglia and cortical ribboning	Positive	3967 pg/ml	Not done	74
54/M	Ataxia, confusion, myoclonus and memory impairment	12	Present	Hyperintensity in basal ganglia	Positive	1788 pg/ml	Not done	55
57/M	Blurred vision, vertigo, ataxia and confusion	14	Present	Hyperintensity in basal ganglia and cortical ribboning	Positive	2976 pg/ml	Not done	58
64/F	Headache, vertigo, confusion, memory impairment and urinary incontinence	14	Present	Hyperintensity in basal ganglia and cortical ribboning	Positive	13,357 pg/ml	Not done	65
56/F	Vertigo, ataxia, insomnia, confusion	12	Present	Hyperintensity in basal ganglia and cortical ribboning	Positive	11,770 pg/ml	Not done	56

*EEG* electroencephalogram, *MRI* magnetic resonance imaging, *CSF* cerebrospinal fluid

## Discussion

This series of cases demonstrates the variety of clinical manifestations that may occur at the onset or during the course of this disease. The age of presentation of sCJD is around 61 years. It is rare in patients younger than 40 years of age [12]. In our series of cases, the average age of symptom onset was 58.8 years, very similar to that reported in the case series of Torres-Ramírez et al., and in the case series of Lolekha et al. The first study reported an average age of onset of 55.8 years in patients with a definitive diagnosis, and 59.6 years in patients with a probable diagnosis [20]. The second study reported an average age of onset of 57 years [21]. The male to female ratio in our patients was 1:1, similar to that reported in other cases reports [9, 12, 13].

Regarding the presentation of disease, it is known that 30% of sCJD cases begin with cognitive or behavioral changes, and 30% begin with focal neurological signs, such as vision loss, cerebellar ataxia, aphasia and motor deficit [8, 9, 12]. In our cohort, 2 cases (33%) started with cognitive/behavioral symptoms, while 4 (66.6%) started with focal neurological signs; 1 case with ataxia, 2 with gait disorders and 1 with vertigo and headache.

Myoclonus represent one of the most common signs of sCJD [15]. Gao et al., showed that they were present in 74.2% of cases when the diagnosis of CJD was probable and in 56.3% when possible [22]. This contrasts with what was found in our patients where myoclonus were present in all patients in advanced stages of the disease. Depression in patients with CJD has also been described in the literature [22]. In our patients, one patient had depression.

Pre-mortem clinical diagnosis of sCJD has been modified over time. The most commonly used diagnostic criteria are the ones proposed by the World Health Organization (Table 2). Those criteria do not take in consideration MRI findings [13]. The most recent criteria have taken into account the great contribution that cerebral MRI has provided in the diagnosis of sCJD. According to current criteria proposed by Zerr and The University of Edinburgh [13, 23, 24] a diagnosis of probable CJD requires the presence of rapidly progressive cognitive impairment and at least 2 of the following 4 characteristics: myoclonus, visual or cerebellar disturbances, pyramidal or extrapyramidal signs, and akinetic mutism. In addition, these clinical criteria must be accompanied by at least one of four paraclinical studies. For diagnosis of possible CJD, rapidly progressive cognitive impairment, at least 2 clinical criteria and a duration of disease less than 2 years are required. Definitive diagnosis is made through histopathological study (Table 3) [23, 24]. According to the study by Zerr et al., the combination of the results of the paraclinical tests reaches a sensitivity of 98% and specificity of 71% [23]. In our series of cases, all of the patients met the clinical criteria established by World Health Organization in 1998, Zerr et al., in 2009 and The University of Edinburgh in 2017. In addition, the whole cohort was positive for 14–3-3 protein in CSF, and 6 out of 6 patients had a high signal abnormalities in caudate and putamen nuclei.

**Table 2:** WHO 1998 criteria for diagnosis of sCJD

Diagnostic Certainty	Characteristic
Definite	Diagnosed standard neuropathological techniques; and/or immunocytochemically
Probable	Progressive dementia
	and at least two out of the following four clinical features
	Myoclonus
	Visual/cerebellar dysfunction
	Pyramidal/Extrapyramidal symtoms
	Akinetic mutism
	And
	Positive EEG
	Or
	Positive 14–3-3
Possible	Progressive dementia
	None of 14–3-3 protein and EEG
	and at least two out of the following four clinical features
	Myoclonus
	Visual/cerebellar dysfunction
	Pyramidal/Extrapyramidal symtoms
	Akinetic mutism
	And
	Duration less than 2 years

EEG: electroencephalogram

**Table 3:** The University of Edinburgh 2017 criteria for diagnosis of sCJD

Diagnostic Certainty	Characteristic
Definite	Progressive neurological syndrome AND Neuropathologically or immunocytochemically or biochemically confirmed
Probable	Rapidly progressive cognitive impairment
	Two or more of A – B – C – D
	And
	Typical EEG (Generalised periodic complexes)
OR	Rapidly progressive cognitive impairment
	Two or more of A – B – C – D
	And
	Typical MRI brain scan (High signal in caudate/putamen on MRI brain scan or at least two cortical regions temporal, parietal, occipital, either on DWI or FLAIR
OR	Rapidly progressive cognitive impairment
	Two or more of A – B – C – D
	And
	Positive 14–3-3
OR	elaProgressive neurological syndrome and positive RT-QuIC in CSF or other tissues
Possible	Rapidly progressive cognitive impairment
	two or more of A – B – C – D
	And duration < 2 years

**Clinical Criteria**

A. Myoclonus

B. Visual or cerebellar problems

C. Pyramidal or extrapyramidal features

D kinetic mutism

*EEG* electroencephalogram, *MRI* magnetic resonance imaging, *DWI* diffusion-weighted imaging, *FLAIR* fluid attenuated inversion recovery, *RT-QuIc* positive real-time quaking-induced conversión, *CSF* cerebrospinal fluid

EEG typically shows periodic sharp wave complexes that repeat every 0.5 to 2 s. These alterations occur in only 60% to 70% of patients and are usually present in advanced stages of the disease [7, 12, 15, 25]. Gao et al., demonstrated in their study that these periodic sharp wave complexes are present in 63.5% of cases and reached 90% in those cases in which myoclonus existed [22]. Zerr et al., have determined that the periodic sharp wave complexes have a sensitivity and specificity of 66% and 74%, respectively [26–28].

The tau protein is released after neuronal damage. Its presence reaches a sensitivity of 81% and a specificity of 85% for the diagnosis of CJD. However, the presence of tau protein, together with the 14–3-3 protein has a positive predictive value of 91% [29]. Wook reported that the specificity of the 14–3-3 protein along with the ratio of total and phosphorylated tau protein (phosphorylated-t/total-t) was 90.62% [30]. On the other hand, it appears that the diagnostic accuracy of tau protein depends on the levels of this protein in CSF [31, 32]. Thus, the LR+ of levels of tau protein > 3000 pg/ml is 10.2 for diagnosis of CJD but with levels of tau protein > 10,000 pg/ml the LR+ is 56.4 [31]. Hamlin et al., studied 420 patients with CJD. They demonstrated that tau protein was superior to 14–3-3 protein as a marker in the diagnosis of CJD [32]. A study by Coulthart et al., included 127 patients with definitive diagnosis of sCJD. They demonstrated that protein levels above 2130 pg/ml allowed for the differentiation of CJD from Alzheimer’s dementia with a sensitivity of 93% and a specificity of 100%. When we studied these markers in the CSF of our patients, we found that three of our patients (50%) had tau protein levels above 10,000 pg/ml and three patients had levels close to or above 3000 pg/ml. All patients had positive 14–3-3 protein in their CSF [31].

Shiga et al. have evaluated the usefulness of DWI MRI in CJD. They studied 36 patients with CJD. Brain DWI MRI abnormalities were found with 92.3% of patients. Moreover, brain DWI MRI abnormalities had a 93.8% specificity [33–36]. Young et al., studied a cohort of patients with probable or defined CJD and the presence of abnormalities in brain DWI and FLAIR MRI. Abnormalities, including the presence of cortical ribboning and alterations in the deep gray matter were present in 68% of patients, affectation of the only cerebral cortex in 24% and of the deep substance of the brain in 5%. In this study, the findings in brain DWI and FLAIR MRI had a sensitivity and specificity of 91% and 95%, respectively [37]. In order to differentiate CJD from other rapidly progressive dementias (RPD) Vitali et al. [34] conducted a study by DWI and FLAIR MRI in 83 patients with CJD. This study showed that the hyperintensity of the gray matter was present in all cases of sCJD with certain regions preferentially involved, but never only limbic regions and rarely in the precentral gyrus. In all sCJD cases with basal ganglia or thalamic DWI hyperintensities, there was associated restricted diffusion. This restriction in diffusion was not seen in the other cases of RPD, in which the hyperintensities of the limbic system were common. The sensitivity and specificity of this study to differentiate sCJD from other RPDs was 96% and 93%, respectively. In our cohort of patients, we found the presence of hyperintensity in the basal ganglia in all patients and the presence of cortical ribboning in 4 of 6 patients.

RT-QuIC is a recently described laboratory technique that provides definitive diagnosis of CJD from CSF samples by detecting PrPSc [15, 38] Orru et al., used RT-QuIC with nasal brushings and showed a sensitivity of 97% and a specificity of 100%. This method is even less invasive than lumbar puncture, which only had 77% sensitivity but 100% specificity when the CSF was tested in the same patients [39]. Therefore, this modern technique should be part of the standard initial testing for CJD. However, this technique is not available in Ecuador.

Definitive diagnosis of CJD is only achieved by histopathological study. A cerebral biopsy gives us adequate histopathological information and is considered the cornerstone in the diagnosis of CJD. However, the frequency of positive test results for the disease in the biopsy is surprisingly low [15]. Brain biopsy should be reserved for cases in which the non-invasive studies have not shown positive results for the disease and the cause of symptoms is not found [15]. The neuropathological characteristics of sCJD at the macroscopic level include different degrees of cerebral and cerebellar atrophy and at a microscopic level the presence of intracytoplasmic vacuolization (spongiform changes) in the gray matter, along with marked neuronal loss and astrocytic gliosis. About 10% of the affected patients present deposition of amyloid plaques [40, 41]. In our cohort, we performed a histopathological analysis in one patient, which demonstrated intracytoplasmic vacuolization, neuronal depopulation and astrocytic gliosis, in which a definitive diagnosis of CJD could be established. The remaining patients were diagnosed with probable CJD.

Ninety percent of patients with prion disease die within the first year of disease onset [9]. The average time from the onset of the symptoms to death in this group was 13 months. The mainstay of treatment is symptomatic and supportive, for example, using clonazepam for the treatment of myoclonus. Otto et al., showed a statistically significant improvement in cognitive function in a group of 28 patients with CJD treated with flupirtine, but this is the only study in the literature to report any symptom improvement with the use of this medication [42]. Future targets of therapy involve preventing the conversion of PrPC to PrPSc [15].

## Conclusion

In conclusion, these are the first case reports of CJD in Ecuador from a third level hospital of Quito. Only in one case, we reach a definitive diagnosis through a pathological study. All other cases had a probable diagnosis. This disease should be considered in individuals older than 50 years of age, with a rapidly progressive dementia associated with myoclonus, visual symptoms, and ataxia accompanied by signs of pyramidal and extrapyramidal dysfunction. Although definitive diagnosis must be histopathological, there are ancillary tests currently available that have allowed us to obtain a diagnosis of the disease.

## Abbreviations

CJD: Creutzfeld jakob disease
CSF: Cerebrospinal fluid
DWI: Diffusion-weighted imaging
EEG: Electroencephalogram
FLAIR: Fluid attenuated inversion recovery
LR: Likelihood ratio
MRI: Brain magnetic resonance imaging
NAA: N-acetyl aspartate
PrP: Prion protein
PrPSc: Prion protein scrapie
RT-QuiC: Positive real-time quaking-induced conversion
sCJD: Sporadic CJD
