# Correction to: Case series of Creutzfeldt-Jakob disease in a third-level hospital in Quito

**DOI:** 10.1186/s12883-018-1088-2

**Published:** 2018-06-12

**Authors:** Germaine Eleanor Torres Herrán, Andrés Damián Ortega Heredia, Braulio Martinez Burbano, Marcos Serrano-Dueñas, María Angélica Ortiz Yepez, Raúl Alberto Barrera Madera, Luis Alfredo Masabanda Campaña, Guillermo David Baño Jiménez, Denny Maritza Santos Saltos, Edgar Patricio Correa Díaz

**Affiliations:** 1 Hospital Carlos Andrade Marín, Av. 18 de Septiembre y Ayacucho, Quito, Ecuador; 2 Universidad Central del Ecuador, Calle Iquique y Sodiro, Quito, Ecuador; 3 Facultad de Medicina de la Pontifica Universidad Católica del Ecuador, Avenida 12 de Octubre y Vicente Ramón Roca, Quito, Ecuador

## Correction

Following publication of the original article [1], Andrés Damián Ortega Heredia requested that his name be corrected from:

Andrés Damián Ortega Herrera

to:

Andrés Damián Ortega Heredia

This change has been actualised by means of this correction article.
